# Peripheral Blood Mononuclear Cells HIV DNA Levels Impact Intermittently on Neurocognition

**DOI:** 10.1371/journal.pone.0120488

**Published:** 2015-04-08

**Authors:** Lucette A. Cysique, William J. Hey-Cunningham, Nadene Dermody, Phillip Chan, Bruce J. Brew, Kersten K. Koelsch

**Affiliations:** 1 Neuroscience Research Australia, Sydney, Australia; 2 The Kirby Institute, UNSW Medicine, UNSW Australia, Sydney, Australia; 3 Peter Duncan Neurosciences Unit, St. Vincent’s Centre for Applied Medical Research, Sydney, Australia; 4 Departments of Neurology and HIV, St. Vincent’s Hospital, Darlinghurst, Sydney, Australia; 5 The University of New South Wales (UNSW), Sydney, Australia; 6 Macquarie University, Sydney, Australia; 7 Queen Elizabeth Hospital, Hong Kong Special Administrative Region, People’s Republic of China; University of Pittsburgh Center for Vaccine Research, UNITED STATES

## Abstract

**Objectives:**

To determine the contribution of peripheral blood mononuclear cells’ (PBMCs) HIV DNA levels to HIV-associated dementia (HAD) and non-demented HIV-associated neurocognitive disorders (HAND) in chronically HIV-infected adults with long-term viral suppression on combined antiretroviral treatment (cART).

**Methods:**

Eighty adults with chronic HIV infection on cART (>97% with plasma and CSF HIV RNA <50 copies/mL) were enrolled into a prospective observational cohort and underwent assessments of neurocognition and pre-morbid cognitive ability at two visits 18 months apart. HIV DNA in PBMCs was measured by real-time PCR at the same time-points.

**Results:**

At baseline, 46% had non-demented HAND; 7.5% had HAD. Neurocognitive decline occurred in 14% and was more likely in those with HAD (*p*<.03). Low pre-morbid cognitive ability was uniquely associated with HAD (*p*<.05). Log_10_ HIV DNA copies were stable between study visits (2.26 vs. 2.22 per 10^6^ PBMC). Baseline HIV DNA levels were *higher* in those with *lower* pre-morbid cognitive ability (*p*<.04), and *higher* in those with no ART treatment during HIV infection 1st year (*p* = .03). Baseline HIV DNA was not associated with overall neurocognition. However, % *ln* HIV DNA change was associated with decline in semantic fluency in unadjusted and adjusted analyses (*p* = .01-.03), and motor-coordination (*p* = .02-.12) to a lesser extent.

**Conclusions:**

PBMC HIV DNA plays a role in HAD pathogenesis, and this is moderated by pre-morbid cognitive ability in the context of long-term viral suppression. While the HIV DNA levels in PBMC are not associated with current non-demented HAND, increasing HIV DNA levels were associated with a decline in neurocognitive functions associated with HAND progression.

## Introduction

HIV-associated neurocognitive disorder (HAND) occurs despite combined antiretroviral therapy (cART) with long-term viral suppression and minimal or no neuropsychiatric confounds[1–3]. In such patients, the main neuropathological process responsible for brain damage remains to be elucidated. One major candidate for HIV-related brain injury is continued immune activation, which may in turn result from HIV persistence despite cART. HIV persistence may cause low-level immune activation via diverse mechanisms, including low-level residual viremia, which is detectable in the majority of HIV-infected individuals on long-term cART by ultrasensitive assays[4]. In this context, it remains unclear whether the persistent immune activation is primarily driven by HIV DNA reservoirs (and persistent low-level viremia) in the systemic compartment versus those in the Central Nervous System (CNS). Two theories are currently present in the literature: 1. HIV-related brain injury is primarily driven by HIV activity (i.e., residual HIV viremia and DNA reservoirs) in the systemic compartment, with activated cells (monocytes/macrophages lineage) trafficking to the CNS to cause activation-induced damage; 2. HIV-related brain injury principally results from HIV reservoirs within brain cells (macrophages, glial cells and astrocytes), which triggers chronic immune activation within the CNS separately from the systemic compartment.

The first theory is more directly testable as peripheral reservoirs are accessible[5]. Indeed, the frequency of HIV DNA in PBMC has been associated with cognitive dysfunction in cART-naïve HIV-infected individuals who have otherwise advanced HIV infection (AIDS) and a high prevalence of current HIV-associated dementia (HAD) (as opposed to milder and non-demented forms of HAND)[6]. In the same cohorts, the authors have shown that HIV DNA levels in PBMC were also associated with cognitive dysfunction in cART-treated subjects who have recently reached undetectable plasma HIV RNA levels[7]. Those participants were not chronic in the sense that their time since HIV RNA suppression in plasma was relatively short (6–12 months) and quite a few individuals still had detectable plasma HIV viral load levels; in other words, the HIV DNA reservoirs in these patients may not have been fully established yet. Furthermore, the same group has presented data to suggest that it is HIV DNA within activated CD14+ monocytes, trafficking to the central nervous system (CNS), which primarily correlates with neurocognitive decline in patients who were cART-naïve[8], and in patients with persistent HAND (84% on cART, 60% with undetectable plasma HIV RNA at baseline)[9]. These relationships are yet to be investigated in the context of chronic treated HIV infection.

The HIV reservoirs may also contribute to the non-demented forms of HAND in particular asymptomatic neurocognitive impairment (ANI) and mild neurocognitive disorder (MND)[10], but the evidence for such a process is less robust than in patients with HAD. Even in studies focusing on the activated CD14+ monocytes, it is not clear how many patients had mild chronic and treated HAND in the study of Kusao et al.[9]. Therefore it is unclear how their results apply to this increasingly common HAND category. Moreover, the effect of HIV duration and long-term clinical stability, which is more typical of well-managed cohorts such as those in Australia[1], has not been rigorously explored. This is important because it also relates to the fundamental nature of the diverse types of HIV DNA reflective of HIV reservoirs: HIV DNA during chronic and treated infection is characterised largely by integrated HIV DNA, while during untreated HIV infection non-integrated HIV DNA is the most common form[11].

In contrast, there is no current method for quantifying HIV DNA reservoirs within the brain[12], and previous studies have relied on CSF HIV RNA and other markers of immune activation in the CNS[13].

The primary aim of our study was to establish the contribution of HIV DNA within PBMCs to HAD and non-demented HAND in chronically HIV-infected patients with long-term viral suppression. The second aim was to determine if a change in these HIV DNA levels over time is associated with declining neurocognition.

## Materials and Methods

### Participants

Baseline cohort characteristics have been published in Cysique et al.[1]. The current study included a subset who had neuropsychological testing at baseline and a visit 18-months later. This subset did not differ in terms of demographics and HIV disease/treatment status compared to the entire cohort. The study included 80 adults with chronic HIV infection (Table 1) enrolled between 2009 and 2011 into the HIV and Brain Aging Research Program, a prospective study investigating the effects of HIV infection on the brain in middle-aged persons. The main criteria for inclusion were: at least 45 years old, stable cART for at least six months, nadir CD4 count equal to or below 350 cells/mm3, known HIV duration equal to or greater than five years, and no active opportunistic disease. Exclusion criteria included a history of neurological disorders predating HIV diagnosis, or psychiatric disorders on the psychotic axis (e.g. schizophrenia); current substance use disorders (within 12 months of study enrolment using a formal psychiatric screen) and being non-proficient in English (see [1,14] for more details).

**Table 1:** Baseline and follow-up study sample demographic, laboratory and clinical characteristics.

	Baseline	Follow-up	p
Age	55.08±7.53	56.74±7.61	<.0001
Education	14.06±2.75	14.11 ± 3.20	.85
Gender (count)	79 males / 1 females	-	
Ethnicity (% Anglo-Australian)	98.7%	-	
Australian standardized Predicted WAIS-III VIQ	108.97±7.22	-	
HIV Risk groups (%MSM)	86.2%	-	
HAND %	53.7%	-	
ANI / MND / HAD %	36.2% / 10.0% / 7.5%	-	
History of HAND %	15.0%	-	
Median Nadir CD4-T cell count (cells/mL)	194	-	
Median Current CD4-T cell count (cells/mL)	556	614	<.0001
Plasma HIV RNA <50 cp/mL	97.5%	87.5%	.08 ^3^
Always undetectable % (vs. not always)	-	85%	
CSF HIV RNA <50 cp/mL (N = 34)	97%	-	
Median HIV duration (years)	19.1	-	
AIDS (CDC 1993) ^1^	67.5%	No new CDC C	
Median Current cART duration (months) ^2^	29	37	
ART treated during 1^st^ year of HIV infection	20.0%	-	
Log(n) HIVDNA in PBMCs	5.21 ± 1.42	5.12 ± 1.40	.45 ^4^

Mean ± SD, otherwise indicated; HAND: HIV-associated neurocognitive disorder; ANI: Asymptomatic neurocognitive impairment; MND; Mild Neurocognitive Disorder; HAD: HIV-associated dementia

^1^No new AIDS defining Illness, 1 single case had a CD4 dropping below 200 cp/mL

^2^88.7% did not change their cART between baseline and follow-up. There was a significant difference between those who changed cART and those who did not in their plasma viral load suppression at follow-up: changed and detectable = 33.3%; did not change and detectable = 9.8%; p<.05.

^3^Log_10_ Plasma HIV RNA were compared using Wilcoxon Signed Rank Test

^4^Pearson correlation between baseline and follow-up Log*(n)* is. 73.

### Ethics

All individuals signed an informed consent before participating in the study. This study was approved by the St. Vincent’s Hospital (08/SVH/90) and The University of New South Wales (08380-08/SVH/90) Human Research Ethics Committees.

### Procedure

#### Neurocognitive examination

Detailed procedures are reported in Cysique et al.[1] and Lane et al.[14]. Briefly, we used a standard neuropsychological test battery covering seven ability domains that is in widespread use for NeuroAIDS research in the U.S.[2]. At follow-up alternate versions of tests were used as appropriate (i.e., Hopkins Verbal Learning Test-Revised and the Verbal Fluency). In addition to the neuropsychological functions’ tests, all participants were assessed with the National Adult Reading Test (NART, 2^nd^ Edition[15]); The HIV Neurobehavioral Research Center Instrumental of Activity of Daily Living (HNRC-IADL)[16], and the Personal Assessment for Own Functioning (PAOFI) [17]. The HNRC-IADL, the PAOFI, clinical history and standard neuropsychological examination scores enabled the classification of each into the three categories of the American Academy of Neurology 2007 HAND criteria: Asymptomatic Neurocognitive Impairment (ANI); Mild Neurocognitive Impairment (MND) and HIV-associated dementia (HAD)[18]. The NART error score and the education level (years) were transformed into an Australian standardized index (with an IQ unit mean of 100 and SD of 15) to reflect pre-morbid cognitive ability (see Cysique et al.[1] for more details).

#### DNA extraction and HIV DNA quantification

Cryopreserved PBMCs were recovered from vapour-phase liquid nitrogen storage, and DMSO washed off by addition of RPMI containing 10% Foetal Calf Serum and centrifugation. DNA was extracted from 3-5x10^6^ PBMCs using the Qiagen AllPrep DNA/RNA kit (Qiagen; Hilden, Germany) according to the manufacturer’s protocol. DNA samples were eluted in DNase/RNase free dH_2_O and stored at -80°C for batched analysis. DNA quantity and purity was assessed using a NanoPhotometer (Implen; California, USA). Yields of DNA averaged 30±11%, corresponding to an average DNA concentration of 67.6±24.1ng/μl. A260/A280 ratios were all between 1.8 and 2.0 indicating a high level of purity.

Total HIV DNA levels were quantified by real-time quantitative PCR (qPCR) using a primer and probe set targeting the *pol* gene (amplicon size = 127bp; nucleotides 2536–2662 of HXB-2 genome), previously optimized for broad coverage of HIV subtype B variants[19]. Oligonucleotide primers (mf299; GCA CTT TAA ATT TTC CCA TTA GTC CTA and mf302; CAA ATT TCT ACT AAT GCT TTT ATT TTT TC) and probes (ri15; CAG **G**A**A** T**G**G **A**TG G and ri16; CTG **T**C**A** A**T**G **G**CC A; bold nucleotides indicate locked nucleic acids) with 6-FAM and BHQ-1 5’ and 3’ modifications respectively, were synthesized by Sigma-Aldrich (Missouri, USA). Amplicon specificity was previously assessed by agarose gel electrophoresis and sequencing (data not shown). All samples were assayed in duplicate and HIV DNA copies determined using a set of plasmid (pNL4-3) standards. Reactions contained; 25μl iQ Supermix (Bio-Rad; California, USA), 1μM mf299 and mf302, 100nM ri15 and ri16, 10μl DNA template, and dH_2_O to a final volume of 50μl.

Calculated HIV DNA copy numbers were normalized for total cellular DNA input using the TaqMan β–actin detection kit (Life Technologies; California, USA). The primers (Forward; CGG AAC CGC TCA TTG CC, and Reverse; ACC CAC ACT GTG CCC ATC TA) and probe (FAM/TAMRA labelled) in this kit target a 289bp conserved region of the β-actin exon 3 [20]. All samples were assayed in duplicate and DNA concentration determined using a set of pooled human DNA standards. Reactions contained; 12.5μl of iQ Supermix, 180nM of Forward and Reverse primers, 120nM probe, 5μl DNA template, and dH_2_O to a final volume of 25μl.

Reactions were performed using an iQ5 real-time PCR Detection system (Bio-Rad; California, USA) and were cycled at; 95°C for 3 minutes; then 45 cycles of 95°C for 15 seconds and 60°C for 1 minute.

Average PCR efficiencies were 93.7±2.8% and 86.6±2.7% (with R^2^ values all >0.99) for the HIV DNA and β-actin qPCR respectively. The limit of detection (LOD) for HIV DNA was assumed to be 1 copy per reaction, which contained on average 676ng of genomic DNA, the equivalent of 108,160 cells. The dynamic range of the total HIV DNA assay as determined by the plasmid (pNL4-3) standard curve was 3,000,000 copies (C_q_ = 19.3±0.22) to 3 copies (C_q_ = 39.8.0±0.91) per reaction. Normalised HIV DNA copies from a positive control (DNA extracted from an HIV-infected individual) analysed in each qPCR assay, were 701±203 copies per 10^6^ PBMC.

### Data analysis

For the analyses we used three main neuropsychological outcomes: 1. Overall neurocognitive performance, defined as the battery-wide demographically uncorrected scaled score (mean of 10 and SD of 3). A higher scale score indicates better performance. 2. Non-demented forms of HAND defined as Asymptomatic Neurocognitive Impartment (ANI), and Mild Neurocognitive Disorders (MND); and HAD categories; 3. Overall change score (regression-based change z-score[21] with a mean of 0 and a SD of 1), which represents neurocognitive performance change across the test battery between baseline and follow-up; and 4. Individual tests change scores, which represents change in specific neurocognitive functions (also regression-based change z-scores).

HIV DNA was log_10_ transformed to approximate the Normal Distribution. Percentage change between HIV DNA at baseline and follow-up was calculated using natural log (*ln*) transformed values [22].

#### Univariate analyses

The associations were investigated with Pearson correlation, chi-square, t-test, or ANOVA as appropriate between baseline variables: Pre-morbid cognitive ability, baseline log_10_ HIV DNA; ANI, MND, HAD; ART during 1^st^ year of HIV; HIV duration (years), CD4+ T cell counts, Central Nervous System Penetrance (CPE) score, and cART duration at baseline (months). We also used same types of analyses for follow-up variables: follow-up log_10_ HIV DNA, and HIV RNA detection status as always undetectable versus not always.

#### Multiple regression analyses: log_10_ HIV DNA and baseline neurocognitive performance

Multiple standard regression model with *baseline* overall neurocognitive performance as the dependent variable and the log_10_ HIV DNA as the primary predictor, adjusted for factors which were associated (*p*<.05) with baseline log_10_ HIV DNA in univariate analyses: ART during 1^st^ year of HIV (yes/no) and pre-morbid cognitive ability in a first step. Model was adjusted for current CD4+ T cell count; age, cART duration, in a second step to account for any residual demographic, main HIV disease or treatment effects. Importantly, ART during 1^st^ year of HIV (yes/no) and baseline HIV duration were highly associated (p<.0001), the models were run without HIV duration to avoid colinearity. The same statistical rationale followed for all other regression analyses.

#### Multiple regression analyses: baseline log_10_HIV DNA and neurocognitive performance change

Multiple standard regression models with overall change score as the dependent variable and baseline HIV DNA as the primary predictor were conducted. The model was adjusted for baseline CD4-T cell count; always undetectable status (yes/no); baseline cART duration and with and without ART during 1^st^ year of HIV. Demographics were not entered in this model as they are corrected for in the overall change score.

#### Univariate and multiple regression analyses: % *ln* change in HIV DNA and neurocognitive performance change

Standard regression model using individual and overall change score with HIV DNA as the predictor and then adjusted for relevant factors as determined in previous models.

Statistical significance was set at the conventional *p*≤.05 (2-tailed). We restricted the overall number of comparisons by selecting only baseline HIV DNA levels as those were highly correlated with the follow-up HIV DNA levels. Moreover, we conducted a phase of univariate analyses to reduce the number of variables in multiple regression analyses. Statistical analyses were conducted using the statistical package JMP 10, 2013 SAS Institute Inc.

## Results

### Baseline neurocognitive status and neurocognitive performance change

At baseline, 46% of the patients had non-demented HAND and 7.5% had HAD. At follow-up, neurocognitive function declined significantly in 14% (as defined in Cysique et al.[1]). Importantly, decline was more likely in those with baseline HAD [ANOVA with Control Dunnett’s (neurocognitively normal global change score = -.06±.06 not different from non-demented HAND at *p*>.95) but different from HAD = -0.55±0.51; *p* = .02].

Low premorbid cognitive ability was uniquely lower in those with HAD [ANOVA with Control Dunnett’s (neurocognitively normal global change score = 109.68± 5.87 not different from non-demented HAND at *p*>.97) but different from HAD = 102.07±9.37; *p*<.05].

Moreover, current HAD was associated with *historical HAD* (**χ** ^2^_(2)_ = 7.2; *p*<.03), as opposed to non-demented HAND (ANI+MND). That is 50% of those with a history of HAD had current HAD, compared to 16% of non-demented HAND, and 8% of neuropsychologically normal.

### Baseline and 18-months HIV DNA

**Fig 1:**
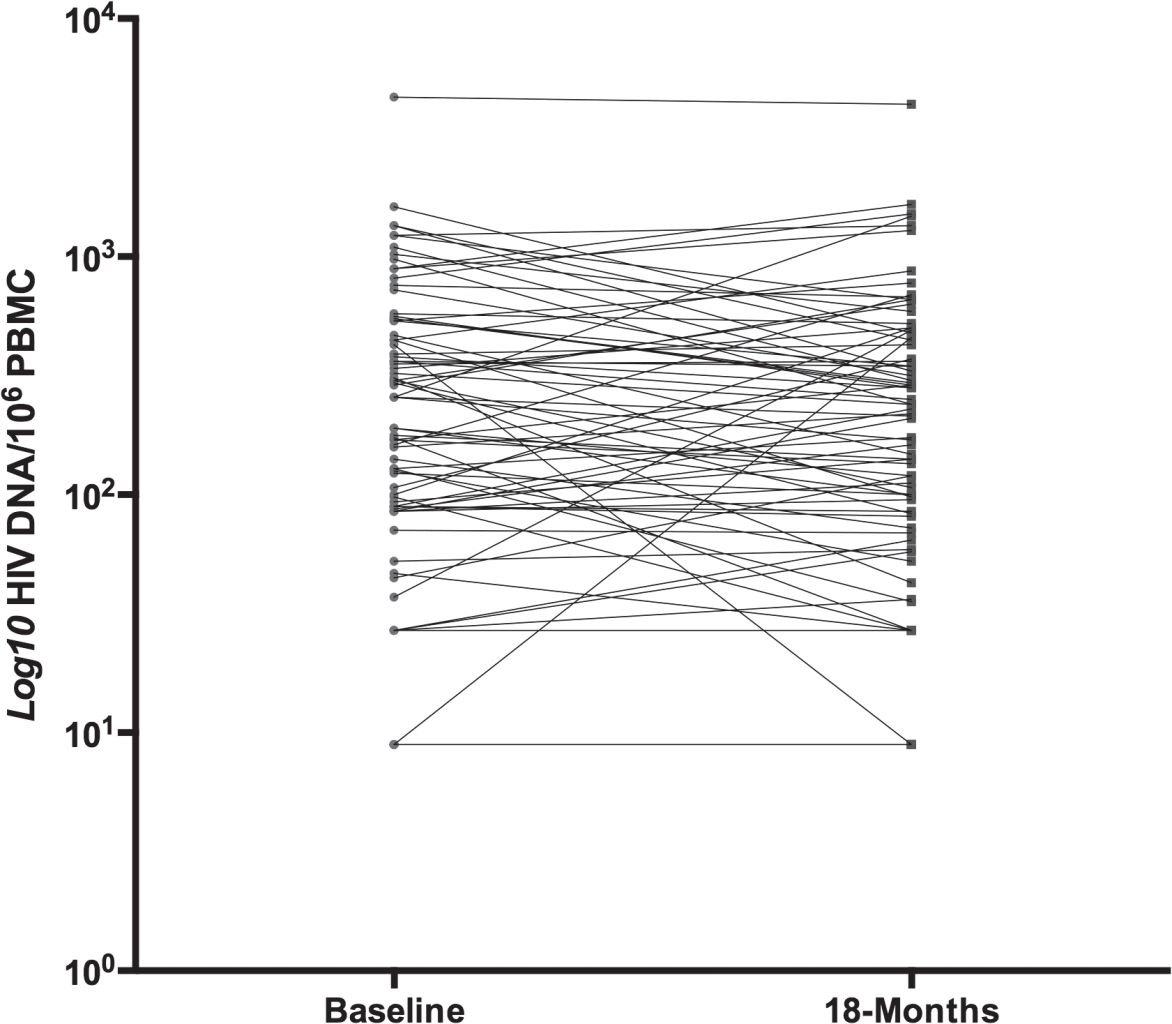
Baseline and follow-up log_10_ HIV DNA per 10^6^ PBMC.

Log_10_ HIV DNA copies were mostly stable between study visits (baseline = 2.26±0.62; follow-up = 2.22±0.61 per 10^6^ PBMC; r = .73; *p*<.0001) (**Fig 1**). Mean % *ln* change between baseline and follow-up was -4.44±26.88.

### Baseline HIV DNA and associations with HIV disease factors

Baseline log_10_ HIV DNA levels in PBMCs were *higher* in those individuals with a *longer* duration of infection (r = .28; *p*<.02). It was also higher in those with *lower* premorbid cognitive ability (r = -.24; *p*<.04). Finally, it was higher in those with no ART treatment during the 1^st^ year of HIV infection (2.33±0.58 vs. 1.97±0.68; t ratio (equal variance) = -2.17; *p* = .03), see **S1, S2, and S3 Figs**. There were no other significant associations.

### Baseline HIV DNA and baseline neurocognitive performance

Multiple regression models showed that baseline HIV DNA levels in PBMCs were not associated with baseline overall neurocognitive performance in unadjusted and adjusted models and it was not associated with HAND (impaired versus unimpaired or neuropsychologically normal, ANI, MND, HAD), see **S4, S5, S6 and S7 Figs**.

### Baseline, follow-up HIV DNA, changes in HIV DNA levels and HIV RNA undetectability (<50cp/mL)

Those individuals with a persistently undetectable plasma HIV RNA during the study period had lower baseline PBMC HIV DNA levels compared to those who did not always have undetectable HIV RNA pVL (2.56±0.41 vs. 2.21±0.63; *p*<.05). The same pattern was present for follow-up HIV DNA although it did not reach statistical significance (2.54±0.59 vs. 2.17±0.57; *p* = .06). There was no relationship between % *ln* change HIV DNA and HIV RNA status (*p*>.60). There were two patients with viral blips/rebounds at baseline (60 and 13000 cp/mL), and 10 patients with blips/rebounds at follow-up (2 patients = 70 cp/mL; four patients = 110–150 cp/mL, three patients = 160–460 cp/mL and one patient = 13000 cp/mL).

### Baseline HIV DNA and neurocognitive performance change

Multiple regression models showed that baseline HIV DNA levels (all p >.40) were not associated with the overall change score in unadjusted and adjusted models.

### Change in HIV DNA and neurocognitive performance change

**Fig 2:**
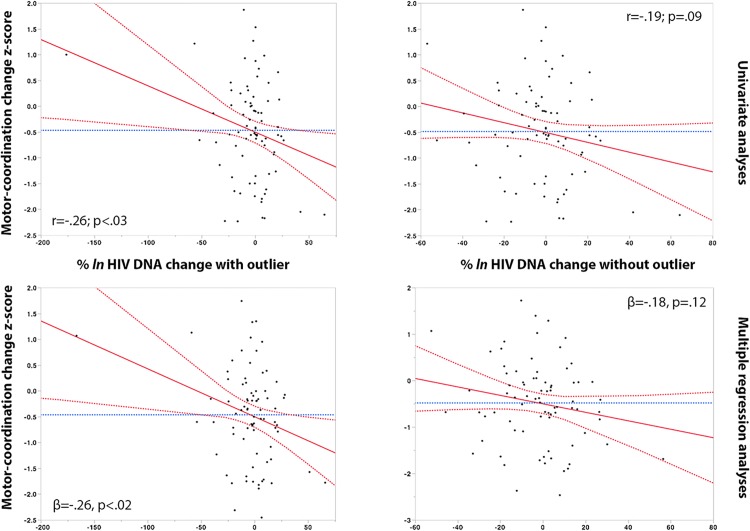
% *ln* HIV DNA change and decline in motor-coordination. Red dashed line: 95% Confidence of interval, red line: Regression mean fit, blue dashed line: set at the mean of Y Leverage Residuals

**Fig 3:**
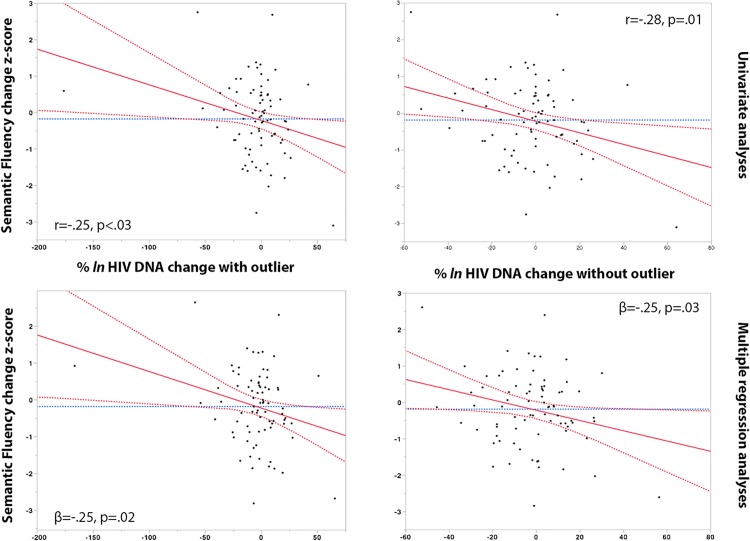
% *ln* HIV DNA change and decline in Semantic Fluency. Red dashed line: 95% Confidence of interval, red line: Regression mean fit, blue dashed line: set at the mean of Y Leverage Residuals

Change in % *ln* HIV DNA levels between study time-points was associated with a decline in motor-coordination (Grooved Pegboard dominant hand) (r = -26, *p*<.03), and semantic fluency (animal category) (r = -25, *p*<.03). In multiple adjusted regression models, the % *ln* change in HIV DNA remained associated with declining motor coordination (ß = -.26, *p*<.02), and declining semantic fluency (ß = -.25; *p* = .02). In control analyses we excluded one HIV DNA change outlier, analyses remained significant for semantic fluency and trended for motor-coordination. Analyses are reported with and without the outlier as further inspection of this data point confirms that it was most likely a real datum (**see Figs 2 and 3**).

## Discussion

Our study was characterized by three main findings: 1. Levels of HIV DNA in PBMC are not associated with overall neurocognitive performance, or HAND clinical categories in chronically HIV-infected adults on long-term suppressive cART. 2. In spite of this, higher levels of PBMC HIV DNA are associated with lower pre-morbid cognitive ability, and the latter is uniquely associated with baseline and historical HAD. This is the first time that an association between pre-morbid ability and HIV DNA levels in PBMC has been observed. 3. While the variation of the HIV reservoir size in PBMC is small over the 18-months period, an increase in reservoir size is associated with decline in neurocognitive functions that have been associated with progression to HAND.

While the magnitude of PBMC HIV DNA plays a definitive role in the occurrence of HAND, this role is largely restricted to HAD (as opposed to the non-demented forms of HAND). This confirms the majority of evidence in regard to the role of PBMC HIV DNA in cART treated HAD [5,23]. Furthermore, the HIV reservoir size has a partial, albeit detectable (including in multiple regression model) and intermittent role (i.e., in the past via pre-morbid ability link, not at baseline but at follow-up) in ongoing HIV-related brain injury. Because HIV replication is mostly suppressed in chronic and treated HIV infection, it may take longer for this residual activation to trigger clinically significant HIV-related brain injury. In any case, our data support the notion that HAD leads to lifelong neurocognitive vulnerability.

Our findings fit with a multi-dimensional model of HIV-related brain injury in which both the peripheral and the CNS HIV reservoirs may have a role. These two reservoirs are possibly dissociated in the case of chronic and treated HIV infection and could have an additive impact on HIV-related brain injury only in some specific instances. Because in most patients with the non-demented form of HAND, peripheral blood HIV DNA content did not play a major role, it is possible that the main neuropathological factor for HIV-related brain injury is restricted to the CNS itself. In contrast, in those with HAD, both reservoirs may play an additive role; hence they lead to greater brain disease severity.

There are key differences between our study cohort, and the cohorts in which the correlation between PBMC HIV DNA levels and HAND (including non-demented forms of HAND) were initially detected [10]. Indeed, their sample mean age was 10 years younger than the current cohort, which is likely to affect the duration of HIV infection, and 42% had undetectable viral load (compared to 97% in our cohort). It is also the patients with HAD who were driving the observed link between HIV DNA and neurocognitive deficits. We computed effect sizes from available mean and SD data in Shiramizu et al. [10] between normal-cognition and HAD (d = 1.6 in entire sample, d = 1.8 in undetectable and d = 1.4 detectable) as opposed to mild forms of HAND versus normal cognition (d = .54 in entire sample, d = .61 = in undetectable and d = .48 in detectable). It is plausible that the medium effect size becomes smaller with increased chronicity of HIV infection and successful treatment and reduced frequency of HAD.

It is therefore important to consider the cohort demographics, clinical and treatment status as these characteristics may have a strong impact on the magnitude of any correlation if present. It is also important to consider that the lower levels of HIV DNA in long-term suppressed patients may make it harder to identify any associations. In this regard, we found that HIV DNA levels in PBMC were mostly stable between study visits, which is consistent with the current understanding of HIV DNA decay during long-term suppressive ART [24]. The residual viremia detected in this cohort occurred for the majority as viral blips, which are typical of chronic and long-term treated HIV-infected patients, and importantly we accounted for this effect in our multiple regression models. Still, patients with undetectable HIV plasma viral load levels during the study period also had lower HIV DNA levels in PBMC, especially at baseline, possibly reflecting the weak link between HIV RNA and DNA in this chronic cohort. Nevertheless, we should acknowledge that ultrasensitive assays, which were not used in this study, might affect the strength of this association.

In agreement with previous studies we found that lower HIV DNA levels in PBMC was associated with early ART initiation (within the 1^st^ year of HIV infection)[25]. Such key historical data was missing from all previous studies in this research area, and therefore it is unclear if differences between the current study finding and other studies may be associated with this effect. We also found that early initiation of ART was strongly associated with duration of HIV infection. This association is likely to reflect that Australian ART guidelines have evolved, and that individuals who have been diagnosed after the 2000s were more likely to receive early ART than those who were diagnosed before then[26]. Duration of HIV infection was also associated with PBMC HIV DNA levels, but this probably represents the mediating effect of early treatment as HIV reservoirs are relatively stable after one year of cART[27].

Our study has both limitations and strengths inherent to the cohort investigated. The HIV epidemic in Australia has been mostly restricted to men who have sex with men, with the majority of those infected receiving long-term suppressive cART, are highly adherent, and have few confounders[1]. This also means that only a few participants had HAD and among those only a few progressed; this may have led to lack of statistical power particularly in the analyses investigating neurocognitive decline. Despite this we still found significant effects, which were maintained in multiple regression analyses.

We chose to measure HIV DNA in PBMCs as a starting point for investigations. PBMC contain the potentially largest contributor to systemic HIV reservoirs, CD4+ T cells, and provide a robust measurement during long-term suppressive cART. Our results demonstrated the robustness of this measurement, as levels were mostly stable over the two time points. Other research has focused on monocyte subsets within PBMC[8], which represent a smaller pool of systemic HIV reservoirs, but may play an important role in neuropathology. While future studies of ours will aim at more specifically addressing this question, we also believe research into the development of technology that can quantify brain HIV reservoirs *in vivo* in chronic and long-term treated HIV-infected persons is warranted[28].

## Conclusion

The role of HIV reservoirs, as reflected by HIV DNA levels in PBMC, appears intermittent and restricted to HAD in the context of long-term suppressive cART. Further investigations into HIV reservoirs and immune activation within the brain will be important to improve our understanding of HIV neuropathogenesis in these types of cohorts. The collection of in depth information on HIV infection and ART history may also assist these studies.

## Supporting Information

S1 Fig Correlation between baseline HIV DNA and pre-morbid cognitive ability. (PDF)

S2 Fig Correlation between baseline HIV DNA and HIV duration. (PDF)

S3 Fig Association between baseline HIV DNA and ART during the first year of HIV infection. (PDF)

S4 Fig No association between baseline HIV DNA and overall baseline neurocognitive performance (univariate analysis). (PDF)

S5 Fig No association between baseline HIV DNA and overall baseline neurocognitive performance (multiple regression analysis). (PDF)

S6 Fig No association between baseline HIV DNA and baseline neurocognitive impairment status. (PDF)

S7 Fig No association between baseline HIV DNA and baseline HAND clinical categories (ANI, MND and HAD). (PDF)
