# Supplementary figures and images for: Peripheral Blood Mononuclear Cells HIV DNA Levels Impact Intermittently on Neurocognition
Source: PLoS One. 2015 Apr 8;10(4):e0120488. doi: 10.1371/journal.pone.0120488 (PMC4390276; doi:10.1371/journal.pone.0120488)

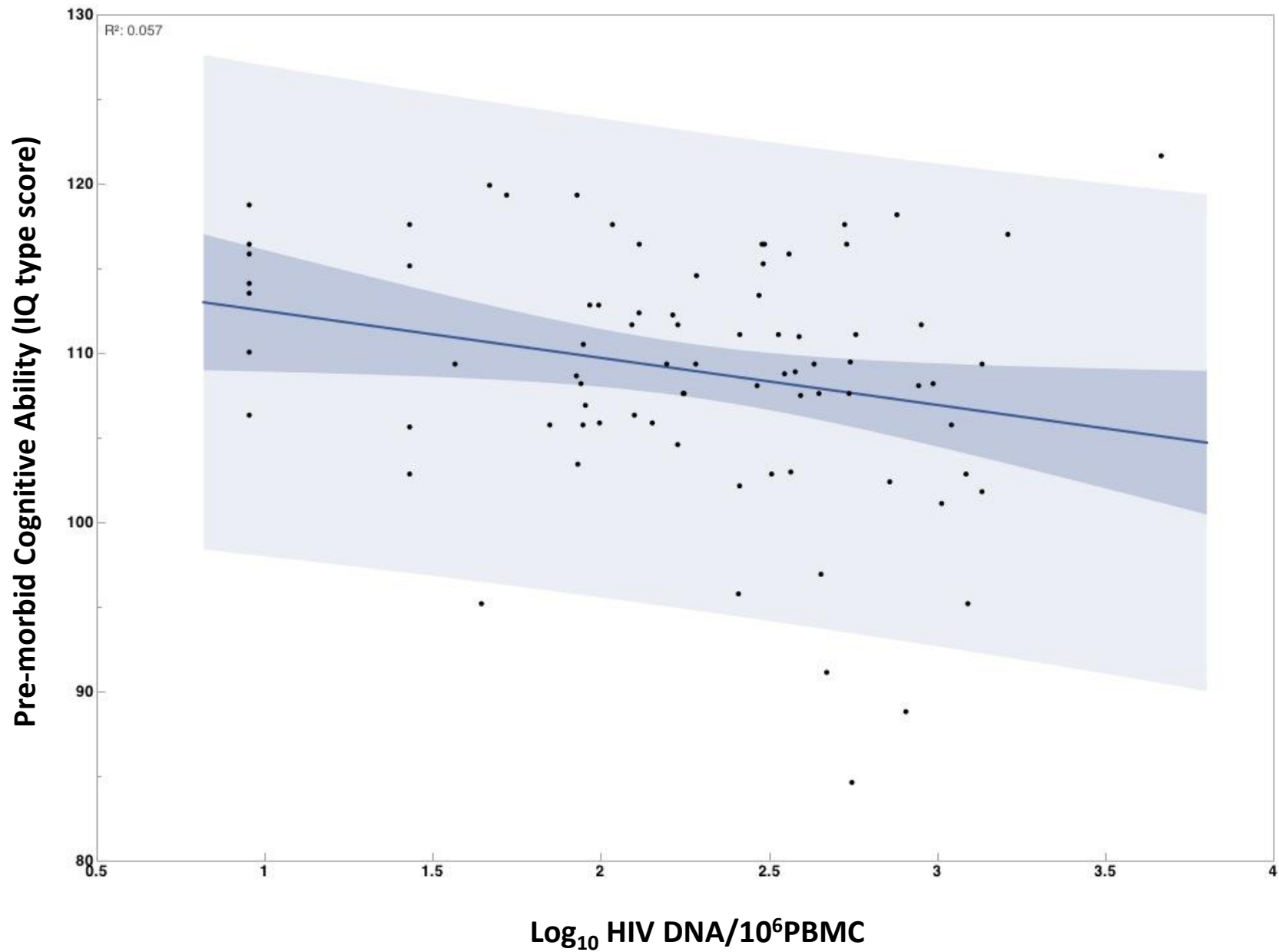

Supplement: S1 Fig — (PDF) [file pone.0120488.s001.pdf]

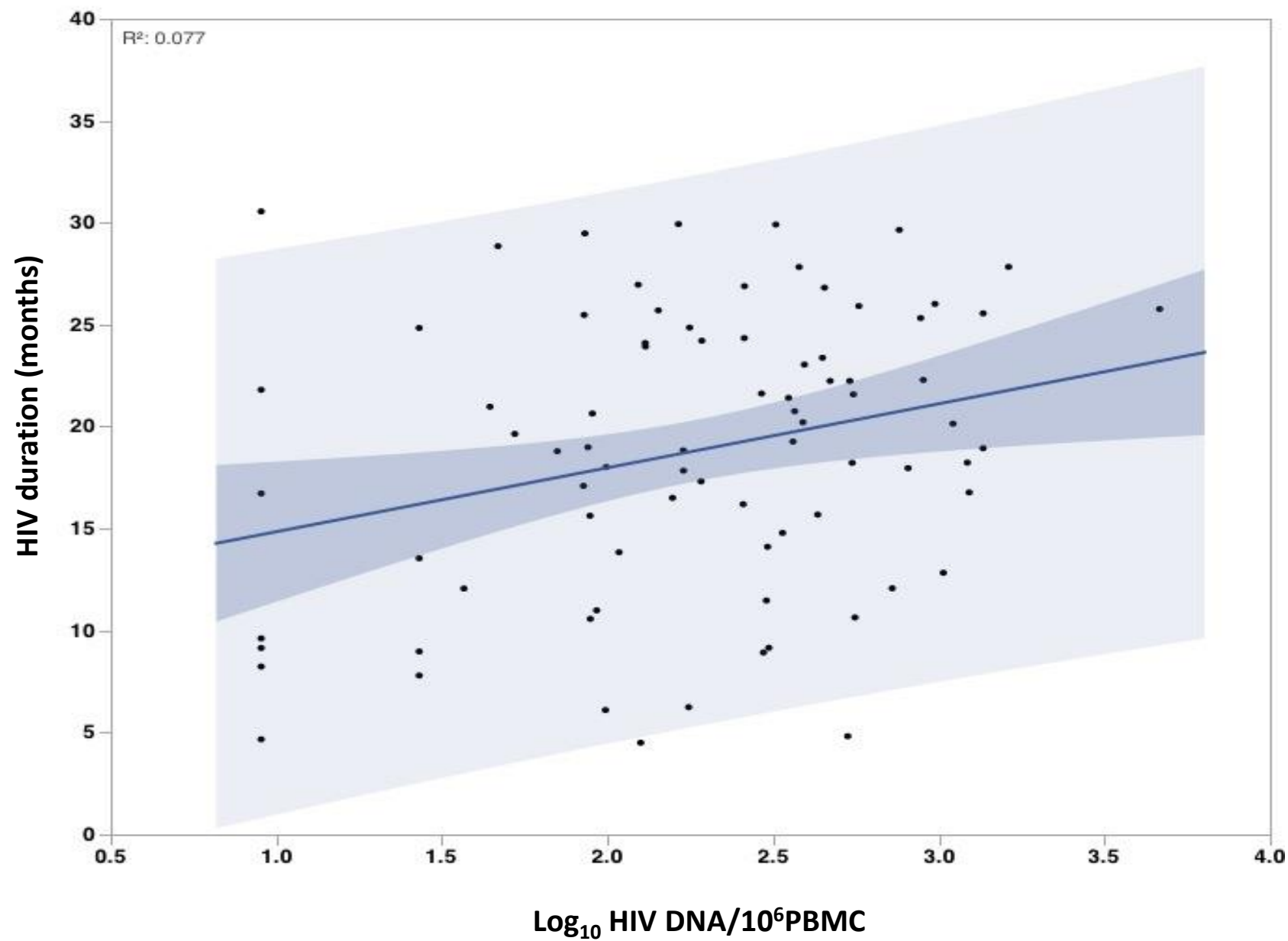

Supplement: S2 Fig — (PDF) [file pone.0120488.s002.pdf]

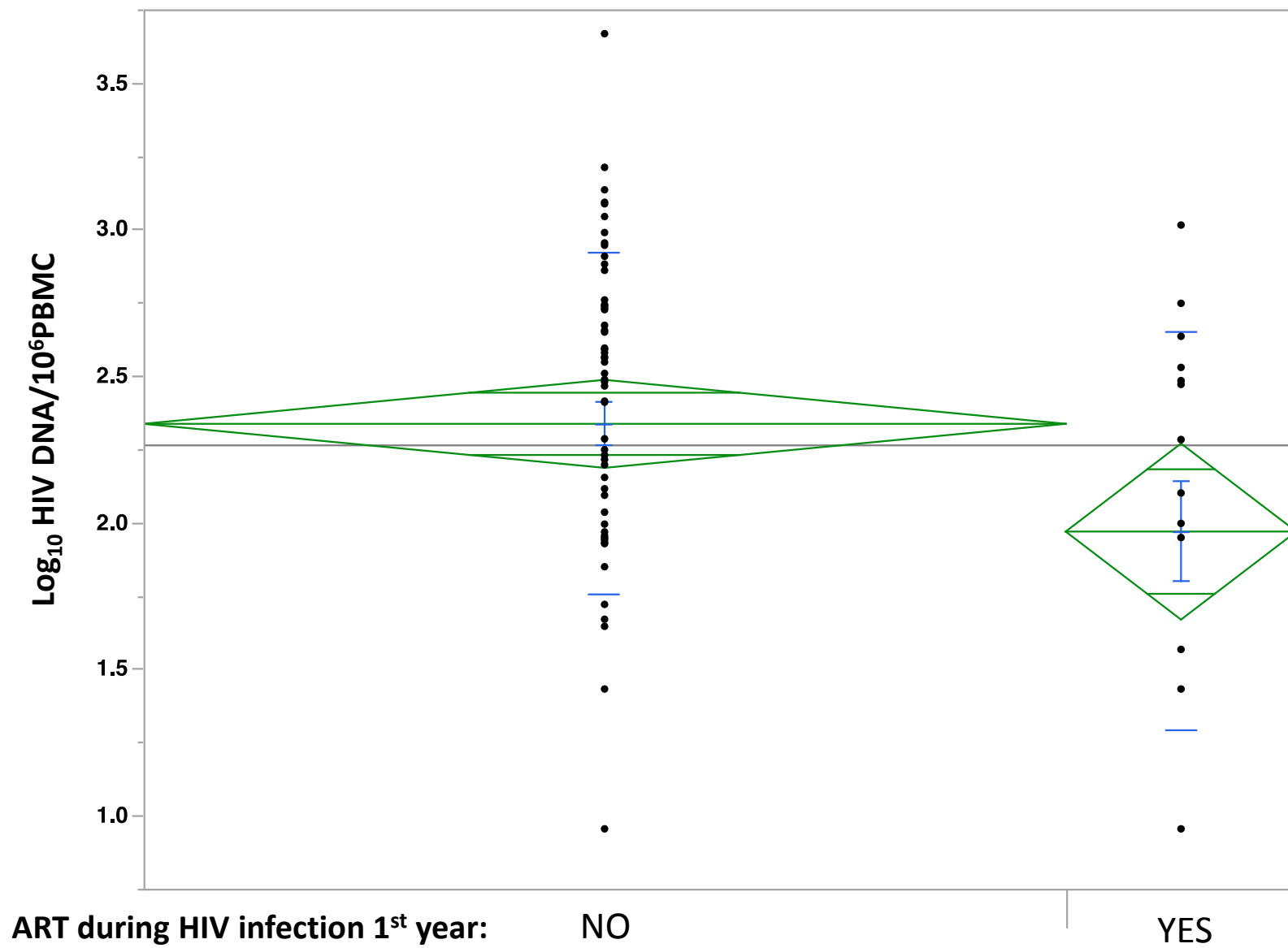

Supplement: S3 Fig — (PDF) [file pone.0120488.s003.pdf]

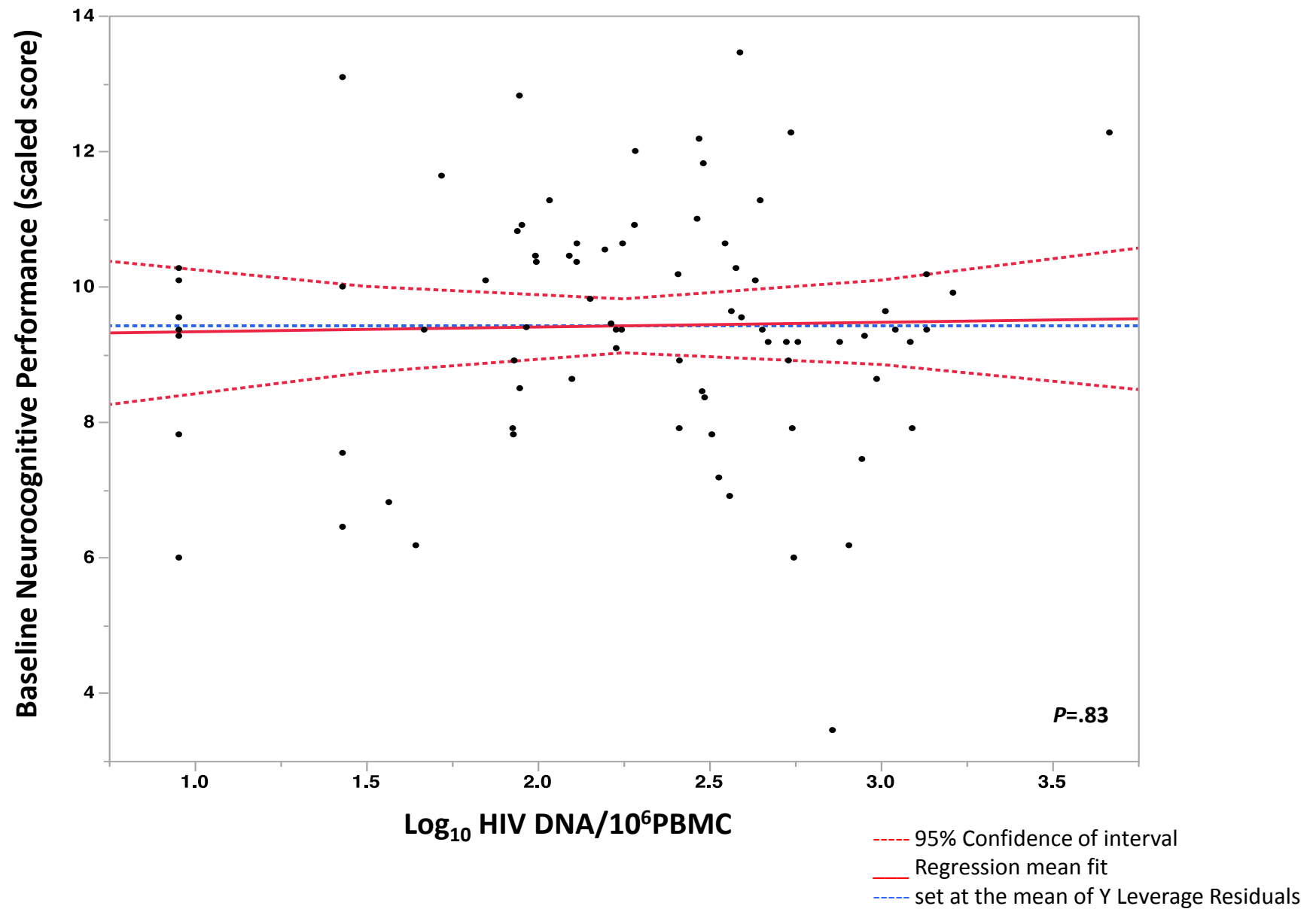

Supplement: S4 Fig — (PDF) [file pone.0120488.s004.pdf]

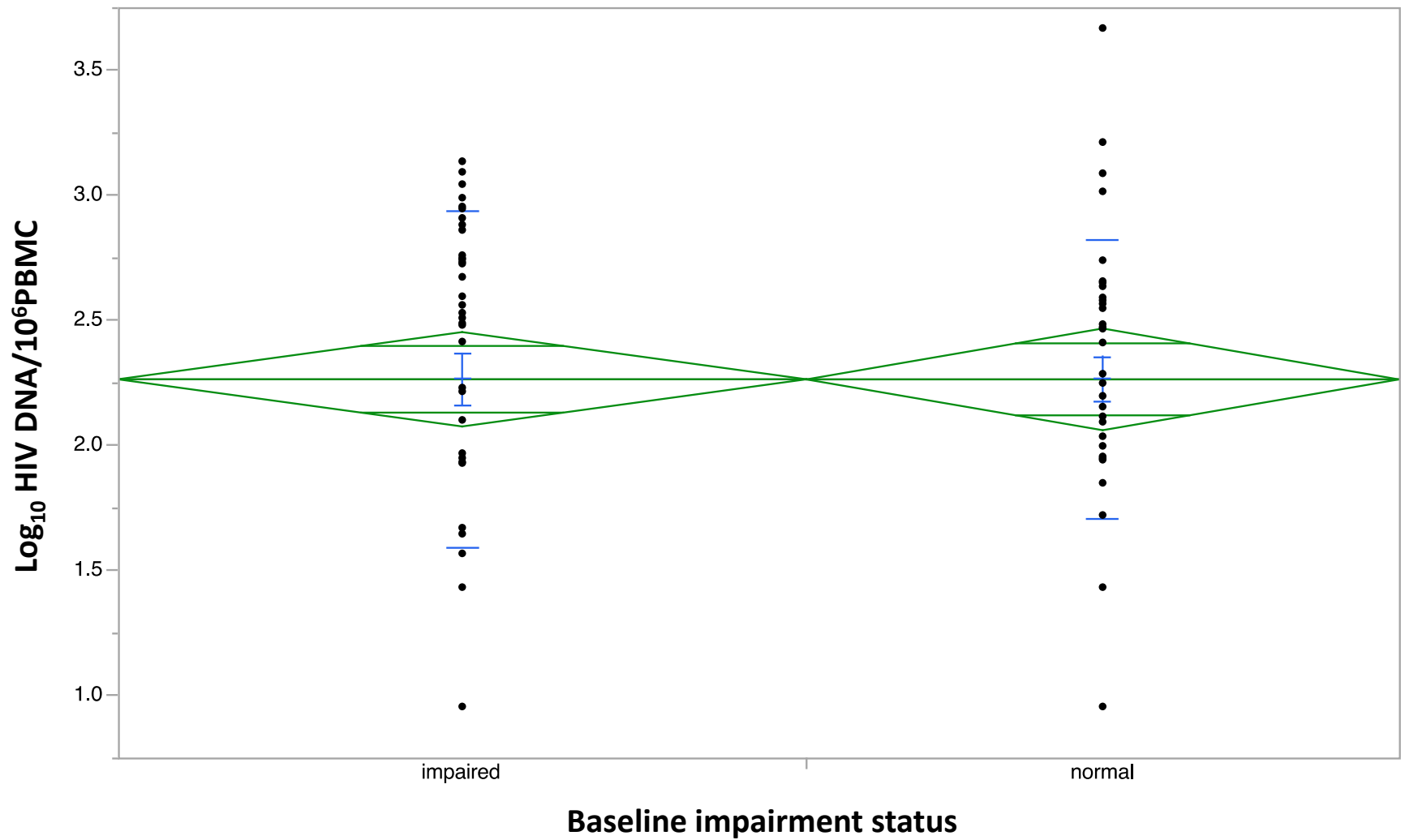

t-test ratio=-.002;p=.99

Supplement: S6 Fig — (PDF) [file pone.0120488.s006.pdf]
