# Supplementary material for: Peripheral Blood Mononuclear Cells HIV DNA Levels Impact Intermittently on Neurocognition
Source: PLoS One. 2015 Apr 8;10(4):e0120488. doi: 10.1371/journal.pone.0120488 (PMC4390276; doi:10.1371/journal.pone.0120488)
Supplement: S5 Fig — (PDF) [file pone.0120488.s005.pdf]

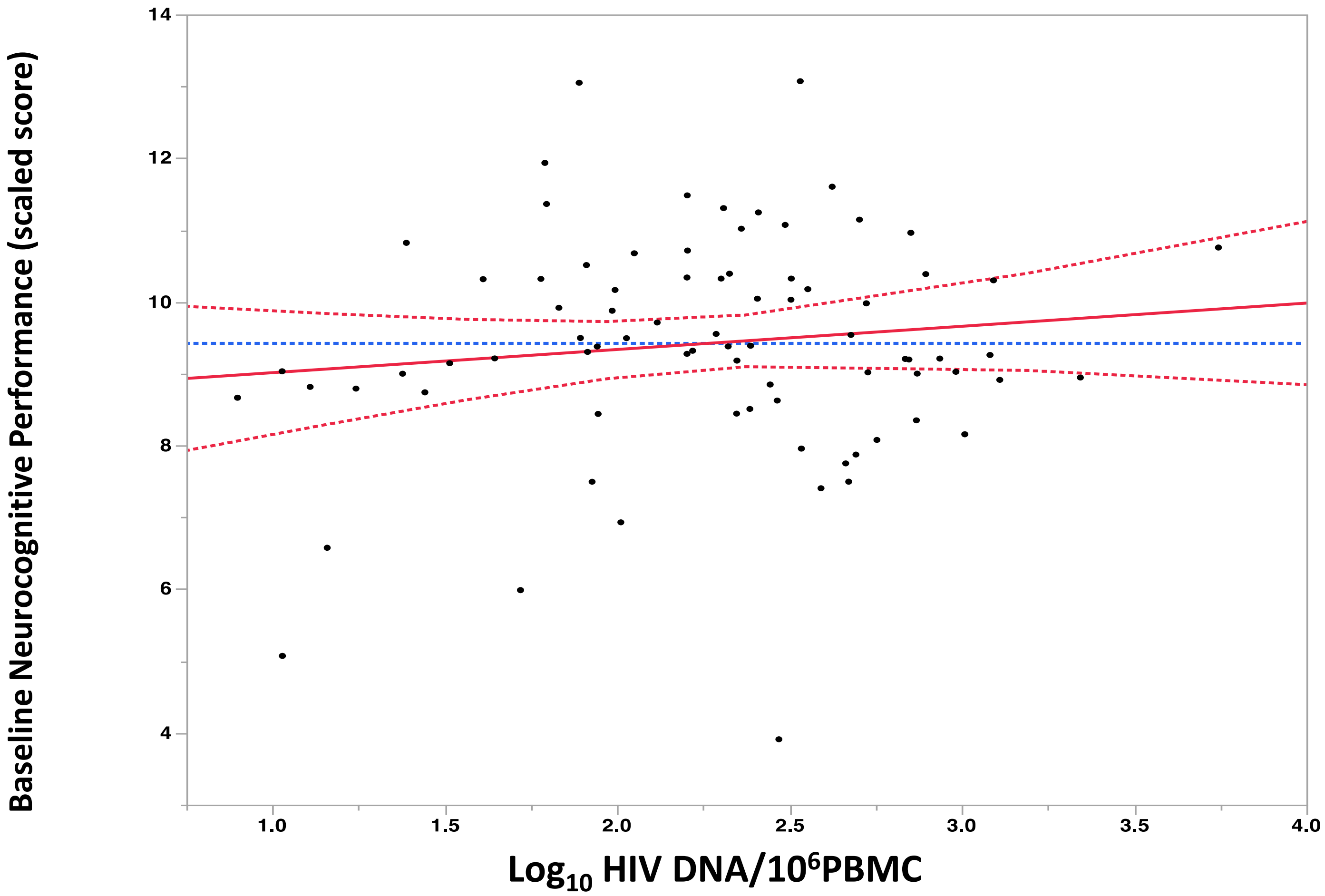

Sorted Parameter Estimates

Term	Estimate	Std Error	t Ratio		Prob>|t|
Pre-Morbid Ability	0.1032606	0.026281	3.93		0.0002*
Age	-0.087482	0.025118	-3.48		0.0008*
Baseline log10 HIV DNA/10^6 PBMC	0.3228454	0.311524	1.04		0.3035
ART During HIV infection 1st year[no]	-0.156667	0.2323	-0.67		0.5022
Baseline cART duration (months)	0.0032084	0.005616	0.57		0.5696
Baseline CD4-T cells count	-0.000355	0.000679	-0.52		0.6024

----- 95% Confidence of interval
____ Regression mean fit
----- set at the mean of Y Leverage Residuals
