# Supplementary material for: Peripheral Blood Mononuclear Cells HIV DNA Levels Impact Intermittently on Neurocognition
Source: PLoS One. 2015 Apr 8;10(4):e0120488. doi: 10.1371/journal.pone.0120488 (PMC4390276; doi:10.1371/journal.pone.0120488)
Supplement: S7 Fig — (PDF) [file pone.0120488.s007.pdf]

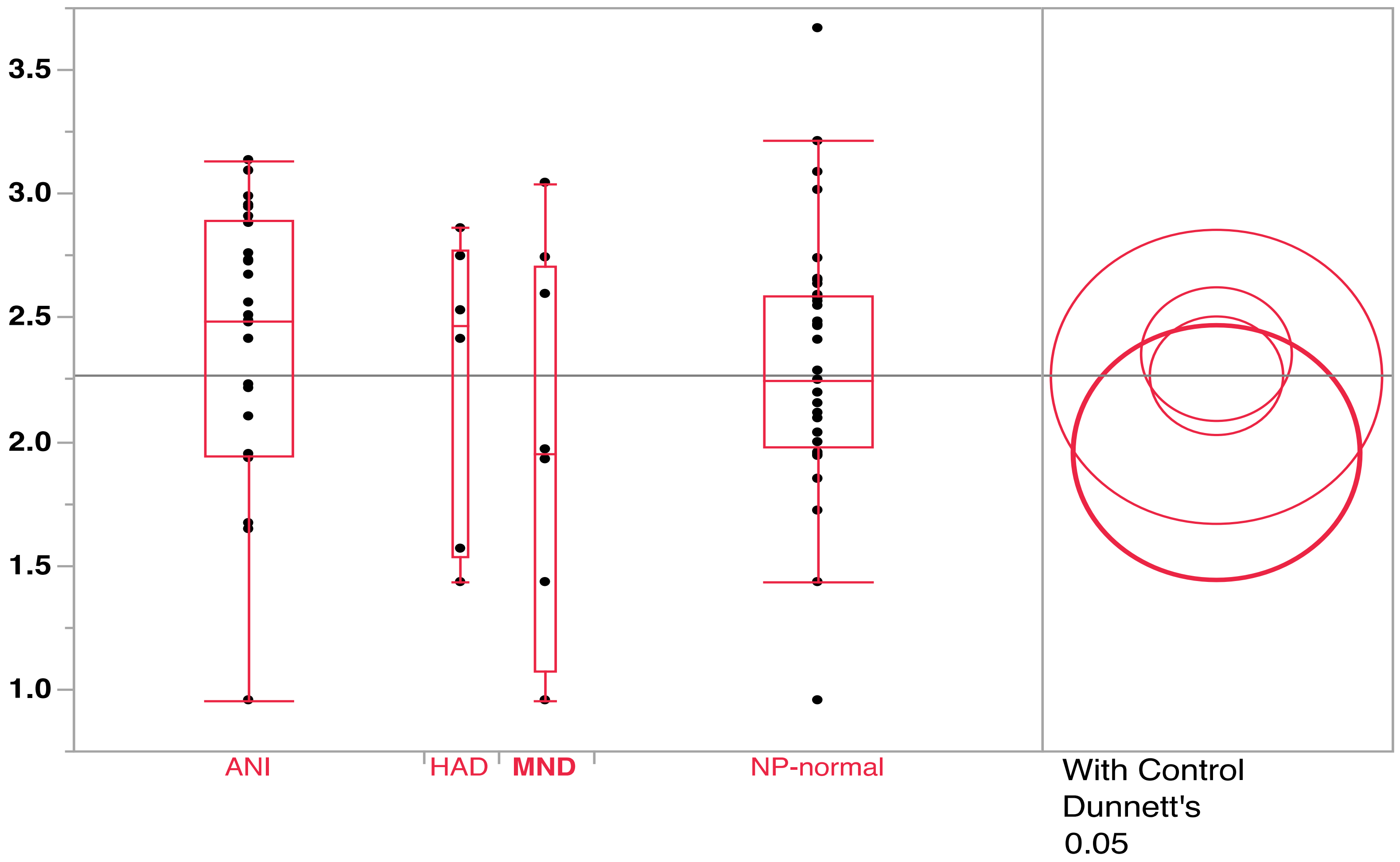

ANOVA F ratio=-0.86; p=.46.
ANI: Asymptomatic Neurocognitive Impairment; MND: Mild Neurocognitive Disorder; HAD: HIV-associated dementia; NP-normal: neuropsychologically normal
